# Treatment of metastatic castration-resistant prostate cancer: review of current evidence and synthesis of expert opinions on radioligand therapy

**DOI:** 10.3389/fonc.2025.1530580

**Published:** 2025-02-25

**Authors:** Darren M. C. Poon, William S. K. Cheung, Peter K. F. Chiu, Daniel H. S. Chung, John B. T. Kung, Daisy C. M. Lam, Angus K. C. Leung, Anthony C. F. Ng, Joe M. O’Sullivan, Jeremy Y. C. Teoh, Philip Y. Wu, Sam K. K. Wu, Philip W. K. Kwong

**Affiliations:** ^1^ Department of Clinical Oncology, State Key Laboratory of Translational Oncology, Sir YK Pao Centre for Cancer, Hong Kong Cancer Institute, The Chinese University of Hong Kong, Hong Kong, Hong Kong SAR, China; ^2^ Comprehensive Oncology Centre, Hong Kong Sanatorium and Hospital, Hong Kong, Hong Kong SAR, China; ^3^ Department of Nuclear Medicine & PET, Hong Kong Sanatorium and Hospital, Hong Kong, Hong Kong SAR, China; ^4^ S.H. Ho Urology Centre, Department of Surgery, The Chinese University of Hong Kong, Hong Kong, Hong Kong SAR, China; ^5^ Department of Clinical Oncology, Queen Elizabeth Hospital, Hong Kong, Hong Kong SAR, China; ^6^ Nuclear Medicine Unit, Department of Diagnostic and Interventional Radiology, Queen Elizabeth Hospital, Hong Kong, Hong Kong SAR, China; ^7^ Department of Clinical Oncology, The Chinese University of Hong Kong, Prince of Wales Hospital, Hong Kong, Hong Kong SAR, China; ^8^ AMO Oncology Centre, Hong Kong, Hong Kong SAR, China; ^9^ Patrick G Johnston Centre for Cancer Research, Queen’s University, Hong Kong, Hong Kong SAR, China; ^10^ Department of Clinical Oncology, Pamela Youde Nethersole Eastern Hospital, Hong Kong, Hong Kong SAR, China; ^11^ Hong Kong Integrated Oncology Centre, Hong Kong, Hong Kong SAR, China

**Keywords:** [^177^Lu]Lu-PSMA-617, genitourinary oncology, metastatic castration-resistant prostate cancer, prostate-specific membrane antigen, radioligand therapy

## Abstract

**Background:**

Despite the boom in the development of cancer management in the last decade, most patients with metastatic prostate cancer (PCa) eventually progress to metastatic castration-resistant PCa (mCRPC) and often require multiple lines of treatment. The treatment landscape of mCRPC has evolved rapidly in recent years, introducing various types of systemic therapies, including taxane-based chemotherapy, androgen receptor pathway inhibitors, bone-targeted radionuclides (e.g., radium-223), immune checkpoint inhibitors, poly(adenosine diphosphate [ADP]-ribose) polymerase (PARP) inhibitors, and radioligand therapies (RLTs) [e.g., a prostate-specific membrane antigen (PSMA) ligand labelled with ^177^Lu].

**Methods:**

To help clinicians navigate the increasingly complex treatment landscape of mCRPC, this article reviews the evidence on different therapeutic regimens from pivotal trials. In addition, it reports on the results of a questionnaire developed and distributed by the Hong Kong Society of Uro-Oncology (HKSUO), with the aim of collecting the perspectives of specialists experienced in the treatment of advanced PCa in Hong Kong with regard to the clinical application of RLT, primarily [^177^Lu]Lu-PSMA-617/analogue therapy.

**Results:**

A total of 43 questionnaire respondents (including clinical oncologists, urologists, nuclear medicine specialists, and medical oncologists) voted on 27 consensus questions divided into eight sections. Consensus or strong consensus (correspondingly ≥75% or ≥90% acceptance for an answer option) was reached for 10 questions. Subsequently, a panel of 13 local and overseas experts coordinated by the HKSUO discussed the voting results and provided further insights into certain questions.

**Conclusion:**

The literature review, the voting results of the questionnaire, and the expert opinions are expected to facilitate better understanding of recent therapeutic advancements and the role of novel RLTs in the treatment of mCRPC among clinicians.

## 1 Introduction

In Hong Kong, prostate cancer (PCa) is the third commonest malignancy in men, accounting for 16.0% of all new cancer cases in men, and is the fourth most frequent cause of male cancer mortality (1). According to international data, 10%–20% of patients with PCa develop castration-resistant PCa (CRPC) within approximately 5 years, with ≥84% of patients having metastases at CRPC diagnosis, whilst 33% of patients with no metastases at diagnosis of CRPC develop them within 2 years (2). Although clinically localised PCa can be effectively treated with radical prostatectomy or radiotherapy with a favourable prognosis (3), metastatic PCa is a complicated, incurable disease that is associated with multiple complications (primarily skeletal-related events caused by bone metastases), worsened quality of life, and substantial casualties (approximately two-thirds of patients die within 5 years after diagnosis) (4, 5).

Androgen deprivation therapy (ADT), which inhibits testosterone production, remains the foundational systemic treatment of metastatic PCa (6). The treatment paradigm shifted from ADT alone to ADT plus one to two upfront agents [an androgen receptor pathway inhibitor (ARPI) ± docetaxel chemotherapy], significantly improving the disease control of metastatic hormone-sensitive PCa (mHSPC) (7–11). However, most patients eventually develop resistance and progress to metastatic CRPC (mCRPC), which often necessitates multiple lines of life-prolonging systemic therapies on top of continuous ADT (6).

In recent years, the treatment landscape of mCRPC has evolved rapidly. In addition to chemotherapy, ARPIs, and bone-targeted radionuclides, a variety of systemic therapies using different platforms have been studied and introduced, including immune checkpoint inhibitors (ICIs), poly(adenosine diphosphate [ADP]-ribose) polymerase (PARP) inhibitors, and radioligand therapies (RLTs) (12, 13). The optimal choice and the sequencing of treatment should be individualised based on a number of considerations, such as the previously received treatment, the presence of actionable biomarkers, the fitness of the patient, and the characteristics of the tumour (12, 13).

To help uro-oncology practitioners navigate clinical decision-making through the increasingly complex treatment landscape of mCRPC, this article reviewed the evidence on the different therapeutic agents from pivotal trials and discussed the results of a questionnaire that collected the perspectives from specialists experienced in the treatment of PCa in Hong Kong with regard to the clinical application of RLT.

## 2 Review of the established therapies for mCRPC

Docetaxel is a conventional chemotherapy for mCRPC based on two historical phase III randomised trials, namely, SWOG 99-16 and TAX 327 (14–16). Cabazitaxel is a more recently developed chemotherapy for patients with mCRPC who progress after docetaxel treatment based on the TROPIC phase III study and the CARD phase IV study (17, 18). Abiraterone and enzalutamide are ARPIs for the treatment of mCRPC. The efficacy and the safety of these agents were demonstrated in phase III randomised trials that included patients with mCRPC who progressed on docetaxel treatment (COU-AA-301 for abiraterone and AFFIRM for enzalutamide) (19, 20) and chemotherapy-naive patients with mCRPC (COU-AA-302 for abiraterone and PREVAIL for enzalutamide) (21, 22). Supported by the ALSYMPCA phase III study, radium-223 is a bone-targeted radionuclide therapy for patients with mCRPC who have symptomatic bone metastases and no visceral metastases, regardless of prior exposure to docetaxel (23). **Table 1** summarises the trial designs and primary outcomes from landmark studies on these established systemic agents.

**Table 1:** Key details of the landmark studies on established systemic therapies for metastatic castration-resistant prostate cancer.

Therapy	Trial setting	Trial^a^	Control	Primary endpoint	HR (95%CI)	*p*-value
Docetaxel–estramustine	First line	SWOG 99-16 (14)	Mitoxantrone–prednisone	mOS: 17.5 *vs*. 15.6 months	0.80 (0.67–0.97)	0.02
Docetaxel q3w^b^	First line	TAX 327 (15, 16)	Mitoxantrone–prednisone	mOS: 19.2 *vs*. 16.3 months	0.79 (0.67–0.93)	0.004
Cabazitaxel^b^	Post-docetaxel	TROPIC (17)	Mitoxantrone–prednisone	mOS: 15.1 *vs*. 12.7 months	0.70 (0.59–0.83)	<0.0001
Cabazitaxel^b^	Post-docetaxel + abiraterone or enzalutamide	CARD (18)	Abiraterone or enzalutamide (alternative to previously used)	mrPFS: 8.0 *vs*. 3.7 months	0.54 (0.40–0.73)	<0.001
Abiraterone–prednisone	Post-docetaxel	COU-AA-301 (19)	Placebo–prednisone	mOS: 15.8 *vs*. 11.2 months	0.74 (0.64–0.86)	<0.0001
Abiraterone–prednisone	First line	COU-AA-302 (21)	Placebo–prednisone	mOS: 34.7 *vs*. 30.3 months	0.81 (0.70–0.93)	0.0033
Enzalutamide	Post-docetaxel	AFFIRM (20)	Placebo	mOS: 18.4 *vs*. 13.6 months	0.63 (0.53–0.75)	<0.001
Enzalutamide	First line (asymptomatic or mildly symptomatic)	PREVAIL (22)	Placebo	mrPFS: NR *vs*. 3.9 months mOS: 32.4 *vs*. 30.2 months	rPFS: 0.19 (0.15–0.23) OS: 0.71 (0.60–0.84)	Both <0.001
Radium-223 + best SOC	Symptomatic bone metastases + no known visceral metastases ± prior docetaxel	ALSYMPCA (23)	Placebo + best SOC	mOS: 14.9 *vs*. 11.3 months	0.70 (0.58–0.83)	<0.001

*CI*, confidence interval; *HR*, hazard ratio; *mOS*, median overall survival; *mrPFS*, median radiographic progression-free survival; *NR*, not reached; *SOC*, standard of care; *q3w*, every 3 weeks.

a CARD was a phase IV study. All other studies were phase III.

b In combination with prednisone or prednisolone.

## 3 Review of novel and emerging therapies for mCRPC

PARP inhibitors, ICIs, and theranostic agents are among the emerging therapies that have been more widely studied and used in patients with mCRPC, particularly after progression on taxane-based chemotherapy and/or ARPI therapy. Several of these agents have been approved for use in selected patients with mCRPC (**Table 2**), whilst others are under research and pending approval.

**Table 2:** Landmark phase III studies and authority-approved indications of novel therapies for metastatic castration-resistant prostate cancer (mCRPC).

Therapy	FDA-approved indication	EMA-approved indication	Trial setting	Trial	Control	Primary endpoint^a^	HR (95%CI)	*p*-value
Olaparib	HRR gene-mutated, post-ARPI mCRPC (24)	*BRCA*-mutated, post-ARPI mCRPC (25)	Post-ARPI, with ≥1 alteration in *BRCA1/2* or *ATM* (cohort A) or ≥1 alteration in 12 other prespecified genes^d^ (cohort B)	PROfound (26)	Abiraterone or enzalutamide (crossover allowed)	Cohort A (≥1 alteration in *BRCA1/2* or *ATM*): mrPFS: 7.4 *vs*. 3.6 months	0.34 (0.25–0.47)	<0.001
Rucaparib	*BRCA*-mutated, post-ARPI + taxane mCRPC^c^ (27)	Not applicable (28)	Post-ARPI, with a *BRCA1*, *BRCA2*, or *ATM* alteration	TRITON3 (29)	Physician’s choice (docetaxel, abiraterone, or enzalutamide)	*BRCA*-mutated subgroup: mrPFS: 11.2 *vs*. 6.4 months ITT group: mrPFS: 10.2 *vs*. 6.4 months	*BRCA*-mutated subgroup: 0.50 (0.36–0.69) ITT group: 0.61 (0.47–0.80)	Both <0.001
Olaparib–abiraterone + prednisone or prednisolone	*BRCA*-mutated mCRPC (30)	Chemo-ineligible mCRPC (25)	First line	PROpel (31)	Placebo–abiraterone + prednisone or prednisolone	mrPFS: 24.8 *vs*. 16.6 months (ITT population, regardless of the HRR gene mutation status)	0.66 (0.54–0.81)	<0.001
Niraparib–abiraterone + prednisone	*BRCA*-mutated mCRPC (32)	*BRCA*-mutated mCRPC (33)	First line, with or without HRR-related gene alterations	MAGNITUDE (34)	Placebo–abiraterone + prednisone	*BRCA*-mutated subgroup: mrPFS: 16.6 *vs*. 10.9 months Overall HRR gene-mutated cohort: mrPFS: 16.5 *vs*. 13.7 months	*BRCA*-mutated: 0.53 (0.36–0.79) HRR gene-mutated: 0.73 (0.56–0.96)	*BRCA*-mutated: 0.001 HRR gene-mutated: 0.022
Talazoparib–enzalutamide	HRR gene-mutated mCRPC (35)	First-line mCRPC (36)	First line (asymptomatic or mildly symptomatic)	TALAPRO-2 (37)	Placebo–enzalutamide	mrPFS: Not reached *vs*. 21.9 months (ITT population, regardless of the HRR gene mutation status)	0.63 (0.51–0.78)	<0.0001
Pembrolizumab	Unresectable/metastatic solid tumours that are MSI-high, dMMR, or TMB-high (≥10 mutations/Mb); progressed on prior treatment; and have no satisfactory alternative treatment options (38)	Not applicable (39)	All patients received ≥1 prior regimen	5 agnostic studies for the MSI-H or dMMR indication (38); KEYNOTE-158 for the TMB-high indication (40)	Not applicable (all studies were phase II)	ORR in patients with MSI-high or dMMR PCa (*n* = 8): 13%; ORR in patients with TMB-high PCa^e^ (*n* = 11): 9%	Not applicable	Not applicable
^177^Lu-PSMA-617 + SOC^b^	PSMA-positive, post-ARPI + taxane mCRPC (41)	PSMA-positive, post-ARPI + taxane mCRPC (42)	PSMA-positive, post-ARPI + taxane mCRPC	VISION (43)	SOC^b^	mrPFS: 8.7 *vs*. 3.4 months mOS: 15.3 *vs*. 11.3 months	mrPFS: 0.40 (99.2%CI = 0.29–0.57) mOS: 0.62 (0.52–0.74)	Both <0.001

*ARPI*, androgen receptor-axis targeted agent; *dMMR*, mismatch repair-deficient; *EMA*, European Medicines Agency; *FDA*, Food and Drug Administration; *HR*, hazard ratio; *HRR*, homologous recombination repair pathway; *ITT*, intention to treat; *mOS*, median overall survival; *MSI*, microsatellite instability; *PSMA*, prostate-specific membrane antigen.

a The median radiographic progression-free survival (mrPFS) outcomes in PROpel were investigator-assessed. The mrPFS outcomes in all other studies were centrally reviewed.

b Standard of care (SOC) included, but was not limited to abiraterone, enzalutamide, bisphosphonates, radiotherapy, denosumab, or glucocorticoid at any dose.

c Under accelerated approval based on the TRITON2 phase II single-arm trial (27).

d Included *BRIP1*, *BARD1*, *CDK12, CHEK1*, *CHEK2*, *FANCL*, *PALB2*, *PPP2R2A*, *RAD51B*, *RAD51C*, *RAD51D*, or *RAD54L*.

e The 11 patients with prostate cancer (PCa) had ≥175 mutations/exome by whole-exome sequencing analysis, which is approximately equivalent to tumour mutational burden (TMB) ≥10 mutations/Mb by the FoundationOne CDx assay (40).

### 3.1 Genomic-based therapies

PARP inhibitors are aimed at blocking the DNA damage repair (DDR) response in tumours with homologous recombination repair (HRR) gene mutations by trapping the PARP bound to DNA single-strand breaks, ultimately killing cancer cells (44). Based on the PROfound phase III trial (26), olaparib monotherapy has been approved by the U.S. Food and Drug Administration (FDA) for the treatment of HRR gene-mutated, post-ARPI mCRPC (24), whilst the European Medicines Agency (EMA) has only approved its use for *BRCA*-mutated, post-ARPI mCRPC (25). The FDA also granted accelerated approval to rucaparib monotherapy for *BRCA*-mutated, post-ARPI, post-taxane mCRPC based on the TRITON2 phase II trial (27). The more recent TRITON3 phase III randomised trial further examined the efficacy and safety of rucaparib (29). However, the EMA has not approved rucaparib for the treatment of mCRPC (28).

The PROpel, MAGNITUDE, and TALAPRO-2 phase III trials investigated olaparib plus abiraterone, niraparib plus abiraterone, and talazoparib plus enzalutamide, respectively, for the treatment of mCRPC in the first-line setting (31, 34, 37). Depending on different indications approved by the FDA and the EMA, these PARP inhibitor–ARPI combinations can be used in *BRCA*-mutated, HRR gene-mutated, or mCRPC patients regardless of the mutation status (25, 30, 32, 33, 35, 36). Based on the available evidence and approved indications from regulatory authorities, the efficacy of PARP inhibition in *BRCA*-mutated mCRPC has been generally established; however, its role in HRR gene-mutated mCRPC remains relatively uncertain.

The use of ICIs for mCRPC remains dubious. No ICIs, as monotherapy or in combination with other agents, have been approved by the EMA for the treatment of mCRPC (39). However, according to the FDA, pembrolizumab is a treatment option for selected patients with mCRPC as its approved indications include all unresectable or metastatic solid tumours that are microsatellite instability (MSI)-high, mismatch repair-deficient (dMMR), or tumour mutational burden (TMB)-high (≥10 mutations/Mb) and that have progressed on prior treatment and have no satisfactory alternative treatment options (38). The limitation, however, is that only minimal numbers of patients with metastatic PCa were included in the clinical trials on pembrolizumab that were reviewed by the FDA (38, 40), probably due to patients with MSI-high, dMMR, or TMB-high PCa being rare, with prevalence rates ranging from 1% to 12% (45–48). Atezolizumab is an emerging ICI candidate for the treatment of mCRPC. The recent CONTACT-02 phase III randomised trial showed that atezolizumab plus cabozantinib (a tyrosine kinase inhibitor) significantly improved the progression-free survival (PFS), but not the overall survival (OS), in mCRPC patients with substantial visceral involvement and prior ARPI use compared with alternate ARPI monotherapy (49).

### 3.2 Radioligand therapies

Prostate-specific membrane antigen (PSMA) is the foundation of the current theranostic approaches to the treatment of mCRPC. Because of its high expression levels in PCa cells, especially those with increased aggressiveness, PSMA serves as an appealing biomarker for imaging and treatment (50). An RLT agent comprises two major components: a PSMA-targeted ligand (either a small molecule, e.g., PSMA-617 and PSMA-I&T, or a monoclonal antibody, e.g., J591) and a radioisotope aimed at destroying the PSMA-expressing tumour cells by emitting β-radiation (e.g., lutetium-177 [^177^Lu]) or α-radiation (e.g., actinium-225 [^225^Ac]) (51, 52).

[^177^Lu]Lu-PSMA-617 is the first PSMA-targeted RLT approved by both the FDA and the EMA for the treatment of patients with PSMA-positive mCRPC who have been treated with an ARPI and a taxane-based chemotherapy (**Table 2**) (41, 42). In the VISION phase III randomised trial of this patient population, [^177^Lu]Lu-PSMA-617 plus standard of care (allowed treatments included, but were not limited to abiraterone and enzalutamide) significantly prolonged both the median radiographic PFS (rPFS) [hazard ratio (HR) = 0.40, 99.2% confidence interval (CI) = 0.29–0.57, *p* < 0.001] and OS (HR = 0.62, 95%CI = 0.52–0.74, *p* < 0.001) compared with standard of care alone (43). The National Comprehensive Cancer Network in the USA endorses the approved indication of [^177^Lu]Lu-PSMA-617 (53), whilst the European Association of Urology recommends RLT for the treatment of pretreated mCRPC patients with one or more metastatic lesions, highly expressing PSMA (exceeding the uptake in the liver) on the diagnostic radiolabelled PSMA positron emission tomography (PET)–computed tomography (CT) scan (54). Similarly, the European Society for Medical Oncology (ESMO) endorses [^177^Lu]Lu-PSMA-617 for the treatment of mCRPC expressing PSMA on PSMA PET and without PSMA non-expressing lesions (ESMO-Magnitude of Clinical Benefit Scale version 1.1, score: 4) (55).

The more recent PSMAfore phase III randomised trial assessed the efficacy of [^177^Lu]Lu-PSMA-617 in taxane-naive patients with PSMA-positive mCRPC who progressed on an ARPI therapy (56, 57). The primary analysis showed that, compared with switching to another ARPI (abiraterone or enzalutamide), treatment with [^177^Lu]Lu-PSMA-617 significantly improved the median rPFS (HR = 0.41, 95%CI = 0.29–0.56, *p* < 0.0001) (56, 57). Several phase I/II studies preliminarily demonstrated the feasibility of combination therapy for PSMA-positive mCRPC using [^177^Lu]Lu-PSMA-617 with another pharmaceutical agent, which included olaparib (the LuPARP study) (58), pembrolizumab [the PRINCE study (59) and the registered trial NCT03805594] (60), and enzalutamide (the ENZA-p trial) (61). The sequential use of [^177^Lu]Lu-PSMA-617 followed by docetaxel for the treatment of mHSPC was also shown positive in the UpFrontPSMA study (62).

The field of PSMA-targeted RLT is rapidly evolving and is becoming an increasingly important armamentarium for the treatment of mCRPC. In addition to [^177^Lu]Lu-PSMA-617, many other theranostic agents, such as [^177^Lu]Lu-PSMA-I&T, [^225^Ac]Ac-J591, and [^225^Ac]Ac-PSMA-617, are being evaluated as monotherapy or as part of a combination therapy, mostly in patients with PSMA-positive mCRPC who progressed on at least an ARPI therapy (52). Preliminary data have shown that [^225^Ac]Ac-PSMA-617, a potent alpha-emitting therapy, may work for patients who have failed [^177^Lu]Lu-PSMA-617 (52). In addition, the co-administration of [^225^Ac]Ac-PSMA-617 and [^177^Lu]Lu-PSMA-617 is being investigated, with the hope of establishing an alpha/beta synergistic treatment effect whilst alleviating toxicities using lower doses of alphas (52).

## 4 Introduction of the questionnaire

Despite the worldwide approval of [^177^Lu]Lu-PSMA-617 for the treatment of mCRPC, including in Hong Kong, several aspects of PSMA-targeted RLT in routine clinical practice, such as the patient selection criteria, the optimal number of cycles, and response monitoring, remain to be determined.

To collect expert opinions among Hong Kong PCa specialists on these aspects of theranostics, the Hong Kong Society of Uro-Oncology (HKSUO) referenced the practice of the Advanced Prostate Cancer Consensus Conference (APCCC) using a modified Delphi process (63, 64) and developed a set of 27 consensus questions in eight sections: i) treatment sequencing in mCRPC; ii) imaging-based patient selection; iii) patient selection by site of metastasis; iv) response monitoring; v) number of cycles; vi) general questions on RLT; vii) impaired bone marrow function; and viii) impaired renal function. The questions were constructed based on the following assumptions: that all treatments and diagnostic procedures are readily available; that there are no treatment contraindications or options to participate in clinical trials; and, unless stated otherwise, recommendations apply only to non-frail patients and patients with prostate acinar adenocarcinoma. The web-based questionnaire was distributed via e-mail to specialists in clinical oncology, medical oncology, nuclear medicine, and urology who treat >10 patients with PCa annually in Hong Kong (including the expert panellists). If physicians did not feel an expert in a specific topic of question, they were provided the option to choose “abstain/unqualified to answer”. For each question, an answer option with ≥75% or ≥90% agreement was considered to reach consensus or strong consensus, respectively. Denominators were based on the number of respondents who voted upon a particular question, excluding those who chose “abstain/unqualified to answer”. At a subsequent roundtable discussion meeting, a panel of 12 local experts (six clinical oncologists, three specialists in nuclear medicine, and three urologists) and one overseas guest panellist (an expert in radiation oncology) discussed the results of the questionnaire and provided further insights into those questions for which no answer options reached the level of consensus.

**Figure 1:**
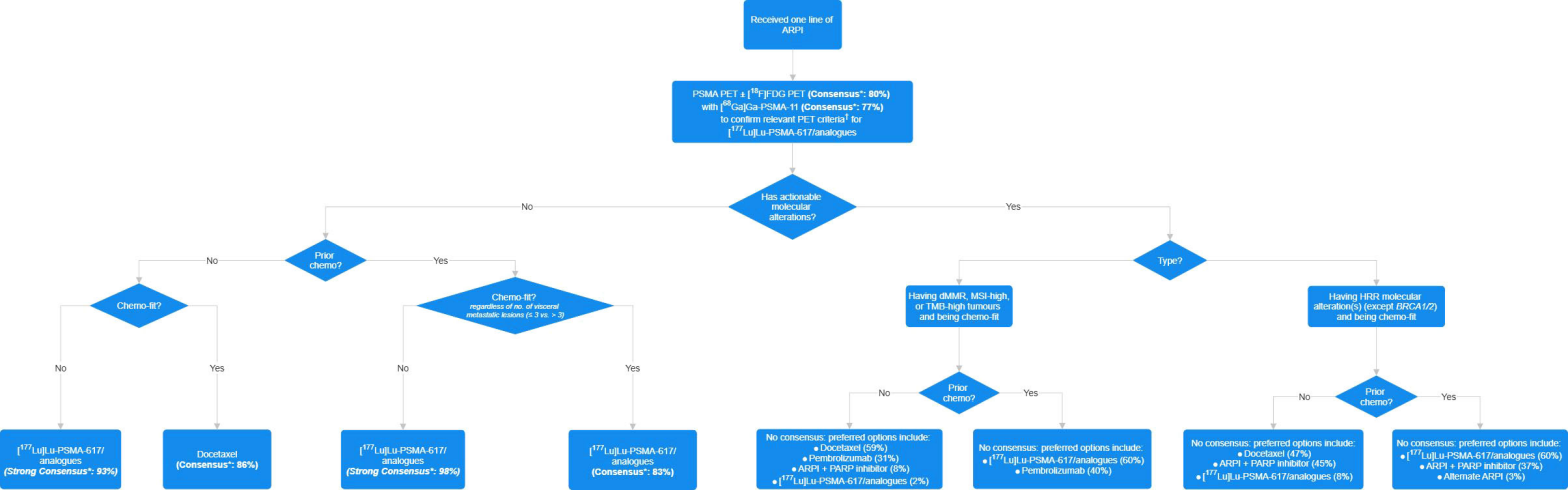
Consensus-based treatment algorithm for patients who received one line of androgen receptor pathway inhibitor (ARPI) therapy for metastatic castration-resistant prostate cancer (mCRPC). *Chemo*, chemotherapy; *dMMR*, mismatch repair-deficient; *FDG*, fluorodeoxyglucose; *HRR*, homologous recombination repair; *MSI*, microsatellite instability; *PARP*, poly (adenosine diphosphate [ADP]-ribose) polymerase; *PET*, positron emission tomography; *PSMA*, prostate-specific membrane antigen; *TMB*, tumour mutational burden. *Asterisk* denotes that an answer option with ≥75% or ≥90% agreement was considered to reach consensus or strong consensus, respectively. *Dagger* indicates that the item is defined as one or more PSMA-positive metastatic lesion ([^68^Ga]Ga-PSMA-11 uptake greater than that of the liver parenchyma in one or more metastatic lesions of any size in any organ system) and no PSMA-negative lesions that meet the following criteria: PSMA uptake equal to or lower than that of the liver parenchyma in any lymph node with a short axis of ≥2.5 cm, in any metastatic solid-organ lesions with a short axis of ≥1.0 cm, or in any metastatic bone lesion with a soft tissue component of ≥1.0 cm in the short axis.

A total of 43 specialists (62% clinical oncologists, 19% urologists, 12% nuclear medicine specialists, and 7% medical oncologists) completed the questionnaire. Consensus or strong consensus was reached for 10 (37%) questions (**Table 3**), and there was no consensus in 17 questions (63%). A treatment algorithm for patients with PSMA imaging-positive mCRPC (**Figure 1**) was derived from the consensuses reached. The full voting results and a summary of the panel discussion are provided below.

**Table 3:** All questions for which consensus or strong consensus was reached.

Section	Question	Answers	Voting results (%/*N*)
1. Treatment sequencing in mCRPC	Q1. For the majority of chemotherapy-fit patients with PSMA imaging-positive mCRPC who meet the relevant PET criteria for [^177^Lu]Lu-PSMA-617/analogues and who have received one line of ARPI and no chemotherapy, what is your preferred treatment option assuming treatments are readily available and there is no actionable molecular alteration?	1) Alternate ARPI	2% (1)
		2) ARPI + PARP inhibitor	0
		**3) Docetaxel**	**86% (36) Consensus**
		4) [^177^Lu]Lu-PSMA-617/analogues	12% (5)
		5) Radium-223	0
		6) Abstain/unqualified to answer	1
	Q2. For the majority of chemotherapy-unfit patients with PSMA imaging-positive mCRPC who meet the relevant PET criteria for [^177^Lu]Lu-PSMA-617/analogues and who have received one line of ARPI and no chemotherapy, what is your preferred treatment option assuming treatments are readily available and there is no actionable molecular alteration?	1) Alternate ARPI	5% (2)
		2) ARPI + PARP inhibitor	2% (1)
		**3) [^177^Lu]Lu-PSMA-617/analogues**	**93% (39) Strong consensus**
		4) Radium-223	0
		5) Abstain/unqualified to answer	1
	Q5. For the majority of chemotherapy-fit patients with PSMA imaging-positive mCRPC who meet the relevant PET criteria for [^177^Lu]Lu-PSMA-617/analogues and who have received one line of ARPI and one line of taxane-based chemotherapy, what is your preferred treatment option assuming treatments are readily available and there is no actionable molecular alteration?	1) Alternate ARPI	0
		**2) [^177^Lu]Lu-PSMA-617/analogues**	**83% (35) Consensus**
		3) Cabazitaxel	17% (7)
		4) Radium-223	0
		5) Abstain/unqualified to answer	1
	Q6. For the majority of chemotherapy-unfit patients with PSMA imaging-positive mCRPC who meet the relevant PET criteria for [^177^Lu]Lu-PSMA-617/analogues and who have received one line of ARPI and one line of taxane-based chemotherapy what is your preferred treatment option assuming treatments are readily available and there is no actionable molecular alteration?	1) Alternate ARPI	0
		2) ARPI + PARP inhibitor	2% (1)
		**3) [^177^Lu]Lu-PSMA-617/analogues**	**98% (41) Strong consensus**
		4) Radium-223	0
		5) Abstain/unqualified to answer	1
2. Imaging-based patient selection	Q9. In the majority of patients that you evaluate for [^177^Lu]Lu-PSMA-617/analogue eligibility, what imaging do you routinely recommend?	1) PSMA PET plus [^18^F]FDG PET (like that in the TheraP study)	7% (1)
		2) PSMA PET and bone scintigraphy (like that in the VISION study)	13% (2)
		**3) PSMA PET and add [^18^F]FDG PET selectively for equivocal cases**	**80% (12) Consensus**
		4) No PSMA PET imaging needed	0
		5) Abstain/unqualified to answer	28
	Q10. In the majority of cases that you evaluate for [^177^Lu]Lu-PSMA-617/analogue eligibility, which PSMA PET ligand do you use routinely?	**1) [^68^Ga]Ga-PSMA-11**	**77% (30) Consensus**
		2) [^18^F]F-PSMA-1007	23% (9)
		3) ^18^F-piflufolastat	0
		4) ^18^F-flotufolastat	0
		5) Abstain/unqualified to answer	4
3. Patient selection by site of metastasis	Q14. In the majority of patients with symptomatic mCRPC meeting the criteria for both treatment with radium-223 and [^177^Lu]Lu-PSMA-617/analogues, which treatment do you recommend?	**1) [^177^Lu]Lu-PSMA-617/analogues**	**91% (39) Strong consensus**
		2) Radium-223	9% (4)
		3) Abstain/unqualified to answer	0
	Q15. For the majority of chemotherapy-fit patients with PSMA imaging-positive, symptomatic mCRPC, with the majority of metastatic lesions in non-visceral sites and with three or fewer visceral metastatic lesions, who meet the relevant PET criteria for [^177^Lu]Lu-PSMA-617/analogues and who have received one line of ARPI and one line of taxane-based chemotherapy, what is your preferred treatment option assuming treatments are readily available and there is no actionable molecular alteration?	1) Alternate ARPI	2% (1)
		2) Docetaxel rechallenge	0
		3) Cabazitaxel	7% (3)
		**4) [^177^Lu]Lu-PSMA-617/analogues**	**86% (36) Consensus**
		5) Radium-223	0
		6) Atezolizumab + cabozantinib	0
		7) EBRT to visceral metastatic lesion(s) then [^177^Lu]Lu-PSMA-617/analogues	5% (2)
		8) EBRT to visceral metastatic lesion(s) then radium-223	0
		9) Abstain/unqualified to answer	1
	Q16. For the majority of chemotherapy-fit patients with PSMA imaging-positive, symptomatic mCRPC, with the majority of metastatic lesions in non-visceral sites and with more than three visceral metastatic lesions, who meet relevant PET criteria for [^177^Lu]Lu-PSMA-617/analogues and who have received one line of ARPI and one line of taxane-based chemotherapy, what is your preferred treatment option assuming treatments are readily available and there is no actionable molecular alteration?	1) Alternate ARPI	2% (1)
		2) Docetaxel rechallenge	0
		3) Cabazitaxel	15% (6)
		**4) [^177^Lu]Lu-PSMA-617/analogues**	**78% (31) Consensus**
		5) Radium-223	0
		6) Atezolizumab + cabozantinib	0
		7) EBRT to visceral metastatic lesion(s) then [^177^Lu]Lu-PSMA-617/analogues	5% (2)
		8) EBRT to visceral metastatic lesion(s) then radium-223	0
		9) Abstain/unqualified to answer	3
5. Number of cycles	Q20. In the majority of patients with response (PSA and/or clinical and/or radiological) to [^177^Lu]Lu-PSMA-617/analogues after four cycles and significant remaining uptake, do you recommend completion of the 6 cycles?	**1) Yes, in the majority of patients**	**90% (36) Strong consensus**
		2) No, only in selected patients	10% (4)
		3) No, in none of the patients	0
		4) Abstain/unqualified to answer	3

*ARPI*, androgen receptor pathway inhibitor; *EBRT*, external beam radiation therapy; *FDG*, fluorodeoxyglucose; *mCRPC*, metastatic castration-resistant prostate cancer; *PARP*, poly(ADP-ribose) polymerase; *PET*, positron emission tomography; *PSA*, prostate-specific antigen; *PSMA*, prostate-specific membrane antigen.

The underlined texts indicate the key patient characteristics concerned in the particular question. The bold texts indicate the results that achieved a consensus or a strong consensus.

### 4.1 Part 1—Treatment sequencing in mCRPC

Recent years have seen drastic transformation of the PCa treatment landscape with treatment intensification in mHSPC coupled with emerging treatment options and modalities. Owing to the increasingly complex treatment consideration and decision-making and the recent availability of RLT as a novel treatment modality in Hong Kong, Part 1 of the questionnaire explored the specialists’ views on the preferred treatment for patients with PSMA imaging-positive mCRPC who meet the relevant PET criteria for [^177^Lu]Lu-PSMA-617/analogues in different clinical settings. Whilst Q1–Q4 focussed on the post-ARPI chemotherapy-naive setting, Q5–Q8 covered the post-ARPI and post-chemotherapy patient population. The presence of specific genetic biomarkers, such as HRR mutations or MSI-high positive status, enables additional treatment options and further complicates the picture. Moreover, patients with advanced PCa can often be frail and unfit for chemotherapy due to comorbidities and/or prior treatment. Therefore, Q2 and Q6 were included to seek local expert perspectives pertinent to chemotherapy-unfit patients. Although the definition of chemotherapy fitness remains unclear, international experts generally consider that patients with a poor performance status, severe hepatic impairment, or intolerance to chemotherapy toxicity from prior exposure are unfit to receive taxane-based chemotherapy (63, 65). Among local experts, consensus or strong consensus was reached for Q1, Q2, Q5, and Q6. In summary, they agreed that, among ARPI-experienced patients with no actionable molecular alterations, [^177^Lu]Lu-PSMA-617/analogues should be considered in those who are unfit or were previously exposed to chemotherapy, whereas docetaxel should be considered in those who are fit and naive to chemotherapy.

**Q1**. For the majority of chemotherapy-fit patients with PSMA imaging-positive mCRPC who meet the relevant PET criteria for [^177^Lu]Lu-PSMA-617/analogues, who have received one line of ARPI and no chemotherapy, and who have no actionable molecular alterations, assuming treatments are readily available, 86% of the respondents preferred docetaxel as the treatment regimen, 12% preferred ^177^Lu-PSMA therapy, and 2% preferred alternate ARPI. There was one abstention. (Consensus for docetaxel)

**Q2**. For the majority of chemotherapy-unfit patients with PSMA imaging-positive mCRPC who meet the relevant PET criteria for [^177^Lu]Lu-PSMA-617/analogues, who have received one line of ARPI and no chemotherapy, and who have no actionable molecular alterations, assuming treatments are readily available, 93% of the respondents preferred ^177^Lu-PSMA as the treatment regimen, 5% preferred alternate ARPI, and 2% preferred ARPI + PARP inhibitor. There was one abstention. (Strong consensus for ^177^Lu-PSMA therapy)

**Q3**. For the majority of chemotherapy-fit patients with PSMA imaging-positive mCRPC who meet the relevant PET criteria for [^177^Lu]Lu-PSMA-617/analogues, who have received one line of ARPI and no chemotherapy, and who have HRR molecular alterations (except *BRCA1/2*), assuming treatments are readily available, 47% of the respondents preferred docetaxel as the treatment regimen, 45% preferred ARPI + PARP inhibitor, and 8% preferred ^177^Lu-PSMA therapy. There were five abstentions. (No consensus for any answer option)

**Q4**. For the majority of chemotherapy-fit patients with dMMR, MSI-high, or TMB ≥10 PSMA imaging-positive mCRPC who meet the relevant PET criteria for [^177^Lu]Lu-PSMA-617/analogues and have received one line of ARPI and no chemotherapy, assuming treatments are readily available, 59% of the respondents preferred docetaxel as the treatment regimen, 31% preferred pembrolizumab, 8% preferred ARPI + PARP inhibitor, and 2% preferred ^177^Lu-PSMA therapy. There were four abstentions. (No consensus for any answer option)

**Q5**. For the majority of chemotherapy-fit patients with PSMA imaging-positive mCRPC who meet the relevant PET criteria for [^177^Lu]Lu-PSMA-617/analogues, who have received one line of ARPI and one line of taxane-based chemotherapy, and who have no actionable molecular alterations, assuming treatments are readily available, 83% of the respondents preferred ^177^Lu-PSMA therapy as the treatment regimen and 17% preferred cabazitaxel. There was one abstention. (Consensus for [^177^Lu]Lu-PSMA-617/analogues)

**Q6**. For the majority of chemotherapy-unfit patients with PSMA imaging-positive mCRPC who meet the relevant PET criteria for [^177^Lu]Lu-PSMA-617/analogues, who have received one line of ARPI and one line of taxane-based chemotherapy, and who have no actionable molecular alterations, assuming treatments are readily available, 98% of the respondents preferred [^177^Lu]Lu-PSMA-617/analogues as the treatment regimen and 2% preferred ARPI + PARP inhibitor. There was one abstention. (Strong consensus for [^177^Lu]Lu-PSMA-617/analogues)

**Q7**. For the majority of chemotherapy-fit patients with PSMA imaging-positive mCRPC who meet the relevant PET criteria for [^177^Lu]Lu-PSMA-617/analogues, who have received one line of ARPI and one line of taxane-based chemotherapy, and who have HRR molecular alterations (except *BRCA1/2*), assuming treatments are readily available, 60% of the respondents preferred [^177^Lu]Lu-PSMA-617/analogues as the treatment regimen, 37% preferred ARPI + PARP inhibitor, and 3% preferred alternate ARPI. There were five abstentions. (No consensus for any answer option)

Taking Q3 and Q7 together, considerable proportions (37% and 45%) of the respondents preferred an ARPI + PARP inhibitor combination in patients with HRR molecular alterations (except *BRCA1/2*) in the chemotherapy-naive and chemotherapy-treated settings, respectively. On the contrary, several panellists at the roundtable discussion cautioned that the efficacy of PARP inhibitors in mCRPC harbouring HRR gene mutations (except *BRCA1/2*) has been modest and is supported by weak evidence; hence, these agents are not preferred when other treatment options are available. Moreover, another panellist expressed preference on prescribing PARP inhibitor monotherapy (PROfound regimen) (26) instead of a combination treatment with ARPI (PROpel regimen) (31) in patients who have immediately progressed from a prior line of ARPI based on current evidence. On the other hand, potential additive effects in DDR inhibition by PARP inhibitors and DNA strand breaking by [^177^Lu]Lu-PSMA-617 are being investigated in the LuPARP study (58).

**Q8**. For the majority of chemotherapy-fit patients with dMMR, MSI-high, or TMB ≥10 PSMA imaging-positive mCRPC who meet the relevant PET criteria for [^177^Lu]Lu-PSMA-617/analogues and who have received one line of ARPI and one line of taxane-based chemotherapy, assuming treatments are readily available, 60% of the respondents preferred [^177^Lu]Lu-PSMA-617/analogues as the treatment regimen and 40% preferred pembrolizumab. There were two abstentions. (No consensus for any answer option)

Considering Q4 and Q8, noticeable proportions (31% and 40%) of the respondents preferred pembrolizumab in patients with MSI-high, dMMR, or TMB ≥10 mCRPC. Indeed, patients with MSI-high, dMMR, or TMB ≥10 PCa are rare, with prevalence rates ranging from 1% to 12% (45–48). In the USA and Hong Kong, pembrolizumab is licensed with a pan-tumour indication that applies for all advanced refractory solid tumours with MSI-high, dMMR, or TMB-high; however, this indication is based on agnostic studies rather than dedicated trials of patients with PCa (38, 66). The pertinent studies only included a total of eight patients with MSI-high or dMMR PCa (38) and 11 patients with PCa with ≥175 mutations/exome by whole-exome sequencing (which is approximately equivalent to ≥10 mutations/Mb by the FoundationOne CDx assay, i.e., the definition of TMB-high) (40). On the contrary, other mCRPC treatments are supported by large registration trials dedicated to patients with mCRPC, such as TAX 327 of docetaxel (N = 1,006) (15, 16), TROPIC of cabazitaxel (N = 755) (17), ALSYMPCA of radium-223 (N = 921) (23), and VISION of [^177^Lu]Lu-PSMA-617 (N = 831) (43).

One panellist shared that, in Hong Kong, BRCA testing in the treatment selection for mCRPC has increasingly been available and practised in recent years. In contrast, clinicians’ perceptions of PSMA PET remain largely restricted to their role in staging PCa rather than their implications for guiding RLT treatment decisions, possibly limiting the clinical use of [^177^Lu]Lu-PSMA-617/analogues for eligible patients.

### 4.2 Part 2—Imaging-based patient selection

In line with the theranostic principle, assessing the avidity of PSMA is constituent to evaluating eligibility for [^177^Lu]Lu-PSMA-617/analogues. Currently, the selection of patients for [^177^Lu]Lu-PSMA-617/analogues based on imaging aspects, such as the definition of a PSMA-positive lesion and PSMA-positive disease, the recommended radioligand for PSMA PET-CT, and the augmentation with [^18^F]FDG PET, remains largely diversified both in clinical practice and in clinical trials.

In the questionnaire, Q9–Q11 asked respondents about their recommended imaging approach, PSMA PET ligand, and the read criteria for assessing patient eligibility for [^177^Lu]Lu-PSMA-617/analogues. On the other hand, although it is described in the literature that PSMA is highly expressed in the tumour tissue of >80% of patients with PCa (67–71), the distribution of PSMA-avid lesions and the fraction of PSMA-negative lesions are unique for each individual patient. Hence, Q12 and Q13 enquired about the treatment strategies for patients with different extents of PSMA-negative lesions. Consensus was reached for Q9 and Q10.

**Q9**. In the majority of patients who are evaluated for eligibility to [^177^Lu]Lu-PSMA-617/analogues, 80% of the respondents recommended PSMA PET and adding [^18^F]FDG PET selectively for equivocal cases, 13% recommended PSMA PET and bone scintigraphy (similar to that in the VISION study), and 7% recommended PSMA PET plus [^18^F]FDG PET (similar to that in the TheraP study). There were 28 abstentions. (Consensus for PSMA PET and adding [^18^F]FDG PET selectively for equivocal cases)

At the APCCC 2024, the panel members reached no consensus on the above question, with 43% choosing PSMA PET and adding [^18^F]FDG PET selectively for equivocal cases, 33% choosing PSMA PET and bone scintigraphy (as in the VISION study), and 24% choosing PSMA PET plus [^18^F]FDG PET (as in the TheraP study) (64). Rather, the local experts reached consensus (80%) on using PSMA PET and adding [^18^F]FDG PET selectively for equivocal cases. At the roundtable discussion, a panellist shared a recent experience rendering the addition of [^18^F]FDG PET for a patient who showed a conflicting clinical picture on the prostate-specific antigen (PSA) level and the PSMA PET-CT image. Moreover, several panellists noted that, considering the cost of ^177^Lu-PSMA therapy, clinicians should consider using dual-tracer PSMA/[^18^F]FDG PET for optimal patient characterisation and treatment consideration. One panellist supplemented that, compared with PSMA PET alone, the addition of [^18^F]FDG PET does not substantially increase the cost or the waiting time in the healthcare setting of Hong Kong, but provides additional information on prognosis and helps to guide treatment decisions. For instance, physicians may opt for localised treatment for a small number of discordant PSMA and [^18^F]FDG lesions pre- or post-[^177^Lu]Lu-PSMA-617/analogues for eligible patients. On the other hand, another panellist pointed out that the clinical efficacy and the safety of [^177^Lu]Lu-PSMA-617 in patients evaluated by PSMA PET without [^18^F]FDG PET in the registration trial, VISION, were superior to that of the comparator arm and have led to the worldwide approval of [^177^Lu]Lu-PSMA-617, indicating that PSMA PET only is sufficient for evaluating patient eligibility for [^177^Lu]Lu-PSMA-617/analogues (41–43). The panellist believes that dual-tracer PSMA and [^18^F]FDG PET potentially exclude patients who may benefit from [^177^Lu]Lu-PSMA-617/analogues. Moreover, there is no direct evidence that patients who demonstrate discordant findings on dual-tracer PSMA and [^18^F]FDG PET would not benefit from [^177^Lu]Lu-PSMA-617/analogues. Excluding patients from [^177^Lu]Lu-PSMA-617/analogues regardless of the extent of discordance might deprive patients from receiving a potentially beneficial treatment. A retrospective analysis demonstrated that, based on the VISION criteria, eligible patients had significant OS and PFS benefits from [^177^Lu]Lu-PSMA-617 compared with ineligible patients; however, when using the TheraP criteria, eligible patients only had a significant PFS benefit compared with ineligible patients (72). The implication is that PSMA PET-CT, instead of dual-tracer PSMA/[^18^F]FDG PET, would be sufficient to identify patients who will benefit from [^177^Lu]Lu-PSMA-617 (72).

Separately, in patients with neuroendocrine PCa who are typically avid for [^18^F]FDG PET but not PSMA PET, the expression of PSMA may occasionally be detected, suggesting the potential role of PSMA PET in exploring the feasibility of PSMA RLT as an additional treatment option upon progression to platinum-based chemotherapy (73). Moreover, [^68^Ga]Ga-DOTATATE PET-CT or somatostatin receptor (SSTR) scintigraphy can be used to evaluate the SSTR expression status of neuroendocrine PCa. Emerging evidence and case reports have demonstrated promising results of [^177^Lu]Lu-DOTATATE therapy for neuroendocrine PCa (74–77). It may be a potential treatment regimen in this group of patients who only have a few effective treatment choices.

**Q10**. In the majority of cases that are evaluated for [^177^Lu]Lu-PSMA-617/analogue eligibility, 77% of the respondents routinely use [^68^Ga]Ga-PSMA-11 as the PSMA PET ligand, whilst 23% routinely use [^18^F]F-PSMA-1007. There were four abstentions. (Consensus for [^68^Ga]Ga-PSMA-11)

[^68^Ga]Ga-PSMA-11 was used as the PSMA PET ligand to evaluate eligibility for [^177^Lu]Lu-PSMA-617/analogues in most randomised clinical trials, such as VISION, PSMAfore, TheraP, and ENZA-p (43, 56, 57, 61, 78). One panellist expressed at the roundtable discussion that the use of [^68^Ga]Ga-PSMA-11 in the VISION trial could have led to the consensus for this ligand. Nonetheless, another panellist pointed out that centres worldwide, especially in Hong Kong, are often faced with shortage of germanium-68/gallium-68 generators and thus increase the utilisation of the fluorine-18-labelled tracer, [^18^F]F-PSMA-1007. A nuclear medicine physician in the panel commented that, upon accumulation of experience on correlating the images with the clinical picture, there are no substantial differences in screening eligibility for [^177^Lu]Lu-PSMA-617/analogues with PSMA PET conducted with [^68^Ga]Ga-PSMA-11 or [^18^F]F-PSMA-1007, as long as the reference organ is correctly identified.

**Q11**. In the majority of patients who are evaluated for [^177^Lu]Lu-PSMA-617/analogue eligibility, 54% of the respondents routinely apply the VISION read criteria for PSMA PET-CT, 23% routinely apply the TheraP read criteria, 14% routinely apply the criteria that patients with all lesions exhibiting higher [^68^Ga]Ga-PSMA-11 (^18^F-piflufolastat or ^18^F-flotufolastat) uptake than the liver are considered PSMA imaging-positive, 6% routinely apply the criteria that patients with all lesions exhibiting higher [^18^F]F-PSMA-1007 uptake than the liver are considered PSMA imaging-positive, and 3% routinely apply the criteria that patients with all lesions exhibiting higher [^18^F]F-PSMA-1007 uptake than the spleen are considered PSMA imaging-positive. There were eight abstentions. (No consensus for any answer option)

In the VISION trial, a PSMA-positive lesion was defined as a disease site with PSMA PET imaging ligand uptake greater than or equal to that in the liver. Eligible patients had one or more PSMA-positive lesions anywhere in the body, and no size criteria were applied on PSMA-positive lesions. Patients were excluded if they have large PSMA PET-negative lymph node(s) with size ≥2.5 cm on the short axis, bone metastasis with soft tissue component with size ≥1.0 cm on the short axis, or solid organ metastasis(es) with size ≥1.0 cm on the short axis (43). In comparison, patients eligible for the TheraP trial had PSMA-positive disease with an SUV_max_ ≥20 at a site of disease and >10 at all other measurable sites of metastatic disease and no sites of metastatic disease with discordant [^18^F]FDG-positive and PSMA-negative findings (78). To pinpoint, the major difference between the PSMA PET/CT-based eligibility criteria of VISION and TheraP lies in whether an SUV_max_ value was applied as a threshold in defining a PSMA-positive lesion.

At the roundtable discussion, a panellist expressed that local practice does not strictly follow the VISION trial when determining PSMA-positive disease as [^18^F]F-PSMA-1007 is used by a number of PSMA PET centres. Nonetheless, the VISION PSMA PET-CT read criteria are principally applied by local experts with the reference organ changed from the liver to the spleen when ^18^F-PSMA-1007 is used instead of [^68^Ga]Ga-PSMA-11. Furthermore, another panellist pointed out that, unlike the clinical trial setting, the specific SUV_max_ thresholds do not form part of the real-life PSMA PET-CT read criteria, but any lesion showing prominent PSMA avidity is regarded as “PSMA-positive”, particularly in the context of limited treatment options for mCRPC patients considering [^177^Lu]Lu-PSMA-617/analogues.

**Q12**. For the majority of chemotherapy-fit patients with mCRPC who have received one line of ARPI and one line of taxane-based chemotherapy, with the majority of lesions being PSMA imaging-positive, with three or fewer PSMA imaging-negative lesions, and with no actionable molecular alterations, assuming all treatments are readily available, 42% of the respondents preferred [^177^Lu]Lu-PSMA-617/analogues as the treatment regimen, 29% preferred external beam radiation therapy (EBRT) to PSMA-negative lesion(s) followed by [^177^Lu]Lu-PSMA-617/analogues, 22% preferred cabazitaxel, 5% preferred cabazitaxel followed by [^177^Lu]Lu-PSMA-617/analogues, and 2% preferred a docetaxel rechallenge. There were two abstentions. (No consensus for any answer option)

Although no answer options reached consensus, a combined 76% of the respondents would prescribe [^177^Lu]Lu-PSMA-617/analogues with or without prior EBRT to PSMA imaging-negative lesions. The radiation oncologists in the panel commented that EBRT to the small number of PSMA-negative lesions followed by systemic therapy such as [^177^Lu]Lu-PSMA-617/analogues for the majority of lesions that are PSMA-positive appears a holistic treatment approach in tackling all cancerous lesions. Preceding EBRT may also provide extra benefit in offering a relief to patients whilst waiting for [^177^Lu]Lu-PSMA-617/analogues to be arranged and initiated. However, it is difficult to conduct a trial to compare between [^177^Lu]Lu-PSMA-617/analogues and the approach of EBRT followed by [^177^Lu]Lu-PSMA-617/analogues. There remains an evidence gap in the role of external radiation in managing oligometastatic disease.

**Q13**. For the majority of chemotherapy-fit patients with mCRPC who have received one line of ARPI and one line of taxane-based chemotherapy, with the majority of lesions being PSMA imaging-positive but with more than three PSMA imaging-negative lesions and no actionable molecular alteration, assuming treatments are readily available, 69% of the respondents preferred cabazitaxel as the treatment option, 11% preferred cabazitaxel followed by [^177^Lu]Lu-PSMA-617/analogues, 8% preferred EBRT to PSMA-negative lesions followed by [^177^Lu]Lu-PSMA-617/analogues, 6% preferred alternate ARPI, 3% preferred [^177^Lu]Lu-PSMA-617/analogues, and 3% preferred a docetaxel rechallenge. There were seven abstentions. (No consensus for any answer option)

When the number of PSMA-negative lesions increases to more than three, a combined 81% voted for cabazitaxel with or without subsequent [^177^Lu]Lu-PSMA-617/analogues, with the most voted option shifting from [^177^Lu]Lu-PSMA-617/analogues to cabazitaxel. Several panellists mentioned that, in addition to the number of PSMA-negative lesions, the sizes and the locations of these lesions, as well as patient preference, should also be considered when making treatment decisions. For instance, [^177^Lu]Lu-PSMA-617/analogues may remain feasible in patients with a few small PSMA-negative lesions confined to the lymph nodes or the lungs.

### 4.3 Part 3—Patient selection by site of metastasis

The bone is the most common site of metastasis among patients with mCRPC: >90% of patients present with bone metastasis over the course of the disease (79). In addition, associated with worse prognosis, 23%–29% of patients with advanced PCa develop visceral metastasis, manifested mainly in the liver and the lungs (80). In particular, liver metastases are often associated with negative PSMA expression (81). Consequently, the following questions sought local expert opinions on the optimal treatment of mCRPC patients with different metastatic sites. Consensus was reached for Q14–Q16, indicating that most respondents recommend [^177^Lu]Lu-PSMA-617/analogues rather than radium-223 in eligible patients with symptomatic mCRPC (Q14) and that [^177^Lu]Lu-PSMA-617/analogues are the preferred treatment for patients with PSMA imaging-positive, symptomatic mCRPC who have received one line of ARPI therapy and one line of taxane-based chemotherapy, with the majority of metastatic lesions in non-visceral sites regardless of the extent (three or fewer vs. more than three lesions) of visceral metastases (Q15 and Q16).

**Q14**. In the majority of patients with symptomatic mCRPC meeting the criteria for both radium-223 and [^177^Lu]Lu-PSMA-617/analogues, 91% of the respondents recommended [^177^Lu]Lu-PSMA-617/analogues and 9% recommended radium-223. There were no abstentions. (Strong consensus for [^177^Lu]Lu-PSMA-617/analogues)

**Q15**. For the majority of chemotherapy-fit patients with PSMA imaging-positive, symptomatic mCRPC, with the majority of metastatic lesions being in non-visceral sites and with three or fewer visceral metastatic lesions, who meet the relevant PET criteria for [^177^Lu]Lu-PSMA-617/analogues, who have received one line of ARPI and one line of taxane-based chemotherapy, and who have no actionable molecular alterations, assuming treatments are readily available, 86% of the respondents preferred [^177^Lu]Lu-PSMA-617/analogues as the treatment regimen, 7% preferred cabazitaxel, 5% preferred EBRT to visceral metastatic lesion(s) followed by [^177^Lu]Lu-PSMA-617/analogues, and 2% preferred alternate ARPI. There was one abstention. (Consensus for [^177^Lu]Lu-PSMA-617/analogues)

**Q16**. For the majority of chemotherapy-fit patients with PSMA imaging-positive, symptomatic mCRPC, with the majority of metastatic lesions in non-visceral sites and with more than three visceral metastatic lesions, who meet the relevant PET criteria for [^177^Lu]Lu-PSMA-617/analogues, who have received one line of ARPI and one line of taxane-based chemotherapy, and who have no actionable molecular alterations, assuming treatments are readily available, 78% of the respondents prefer [^177^Lu]Lu-PSMA-617/analogues as the treatment regimen, 15% preferred cabazitaxel, 5% preferred EBRT to visceral metastatic lesions followed by [^177^Lu]Lu-PSMA-617/analogues, and 2% preferred alternate ARPI. There were three abstentions. (Consensus for [^177^Lu]Lu-PSMA-617/analogues)

### 4.4 Part 4—Response monitoring

Being a novel class of treatment in PCa, the guidelines for post-RLT follow-up are inconsistent and not standardised. Part 4 of the questionnaire surveyed the opinions of the specialists on the approach to monitor the response towards RLT, including the imaging timing and modality, the impact on subsequent treatment decisions, and the clinical implications of different response indicators, such as PSA. No consensus was reached for any question in this part.

**Q17**. In the majority of patients on treatment with [^177^Lu]Lu-PSMA-617/analogues, with regard to early PSA rise or the lack of PSA drop within the first 12 weeks of initiating treatment, 67% of the respondents interpreted this as clinical progression only in selected patients, 21% interpreted this as clinical progression in the majority of patients, and 12% did not interpret this as clinical progression. There were no abstentions. (No consensus for any answer option)

The Prostate Cancer Clinical Trials Working Group 3 (PCWG3) recommended recognising that a favourable effect on PSA may be delayed for ≥12 weeks after treatment initiation; hence, clinicians can ignore early rises (prior to 12 weeks) in determining the PSA response and plan to continue through early rises for a minimum of 12 weeks, unless other evidence of progression are present (82). Among local experts, a combined 79% considered that early PSA rises or the lack of PSA drop does not imply clinical progression in the majority of patients. During the roundtable discussion, one panellist shared that a PSA flare, in fact, occurs with other PCa treatments as well, such as abiraterone and chemotherapy. Whilst the PSA is rising upon progression from the previous line of treatment, it takes time for the effect of the subsequent line of treatment to kick in. To ascertain disease progression during treatment with [^177^Lu]Lu-PSMA-617/analogues, additional factors, such as the duration and the rate of PSA rise, the liver function, the clinical condition of the patient, and radiologic changes on magnetic resonance imaging (MRI) or CT should be accounted for.

In addition, in a post-hoc analysis of the phase III VISION trial, the magnitude of a PSA decline up to 12 weeks with [^177^Lu]Lu-PSMA-617 plus standard of care was strongly associated with prolonged rPFS and OS, as well as delayed worsening of health-related quality of life in patients with progressive, PSMA-positive mCRPC (83).

Besides PSA, imaging serves as an indispensable component in monitoring the response towards ^177^Lu-PSMA.

**Q18**. In the majority of patients on treatment with [^177^Lu]Lu-PSMA-617/analogues, to monitor response in the absence of clinical progression, 53% of the respondents recommended imaging after cycle 4, 37% recommended imaging after cycle 2, and 10% did not recommend imaging. There were three abstentions. (No consensus for any answer option)

**Q19**. In the majority of patients on treatment with [^177^Lu]Lu-PSMA-617/analogues, to monitor response, 55% of the respondents recommended PSMA PET-CT [no intravenous (iv) iodine contrast], 36% recommended PSMA PET plus diagnostic CT (with iv iodine contrast), 7% recommended post-[^177^Lu]Lu-PSMA-617/analogue single-photon emission CT (SPECT)-CT, and 2% recommended conventional imaging. There was one abstention. (No consensus for any answer option)

Imaging for response monitoring in randomised trials of [^177^Lu]Lu-PSMA-617 was largely varied.

In the VISION trial, after the fourth cycle of [^177^Lu]Lu-PSMA-617, the investigator may further administer two additional cycles if the patient fulfilled all of the following criteria: 1) showed evidence of response (i.e., radiological, PSA, or clinical benefit); 2) had signs of residual disease on CT with contrast/MRI or bone scan; and 3) has shown good tolerance to [^177^Lu]Lu-PSMA-617 treatment. In addition, radiographic imaging for tumour assessments was conducted using CT with contrast/MRI or bone scan. Of note is that PSMA PET-CT or SPECT-CT was not conducted during the study treatment (43).

In comparison, in the TheraP trial (73), SPECT-CT imaging encompassing the neck, chest, abdomen, and pelvis was performed 24 (±4) hours after (every) administration of [^177^Lu]Lu-PSMA-617. [^177^Lu]Lu-PSMA-617 may be suspended in the case of exceptional response, i.e., a marked reduction in uptake at all sites of disease with minimally avid or non-PSMA-avid disease on the 24-h post-[^177^Lu]Lu-PSMA-617 SPECT-CT. Patients who have received less than six doses of [^177^Lu]Lu-PSMA-617 may be re-treated with [^177^Lu]Lu-PSMA-617 upon disease progression with symptomatic disease from PCa, PSA progression by PCWG3 recommendation, or radiological progression. Moreover, radiographic response assessment was conducted with CT and bone scan. Of note is that PSMA PET-CT was not conducted during the study treatment.

Furthermore, in the phase II randomised ENZA-p trial, [^68^Ga]Ga-PSMA-11 PET-CT was repeated after two cycles of [^177^Lu]Lu-PSMA-617 (61). Patients who showed adequate volume and intensity of residual PSMA-avid disease for [^177^Lu]Lu-PSMA-617 and met all the safety criteria would proceed with cycles 3 and 4. Otherwise, [^177^Lu]Lu-PSMA-617 would be withheld. In addition, SPECT-CT was performed 24 (±4) hours after (every) administration of [^177^Lu]Lu-PSMA-617. Response was assessed with CT or MRI and whole-body bone scan, with the possible use of PET-CT as well.

Among the questionnaire respondents, a combined 90% recommended imaging for response monitoring during treatment with [^177^Lu]Lu-PSMA-617/analogues in the absence of clinical progression. Several panellists expressed consistent opinions, with the majority of respondents adopting imaging after four cycles of [^177^Lu]Lu-PSMA-617/analogues to satisfy patients’ wish to know the treatment response and/or to determine the presence of remaining PSMA uptake and, thus, whether to continue with the two remaining cycles, resembling the practice in VISION. One panellist commented that, imaging after cycle 2 is typically too early and unnecessary; however, if a pressing clinical need emerges, e.g., bone pain or persistent rise in PSA, imaging may be considered earlier to provide further information for clinicians and patients alike to decide whether to continue [^177^Lu]Lu-PSMA-617/analogues.

Despite response assessment by means of conventional imaging in randomised clinical trials of [^177^Lu]Lu-PSMA-617, as well as the uncertainty on how to interpret the PSMA PET findings in response to [^177^Lu]Lu-PSMA-617/analogues, a majority of questionnaire respondents preferred PSMA PET-CT without iodine contrast for monitoring the response to [^177^Lu]Lu-PSMA-617/analogues. The nuclear medicine specialists in the panel articulated that such preference was due to the superior physical characteristics of PSMA PET-CT compared with SPECT-CT, in addition to the information on functional uptake sizes that a PSMA PET-CT can provide.

In light of the lack of consensus and guidance on interpreting PSMA PET-CT after [^177^Lu]Lu-PSMA-617/analogues, it is hoped that the ENZA-p study could shed light on the interpretation of interim PSMA PET during [^177^Lu]Lu-PSMA-617 treatment and the patient outcomes to the adaptive dosing approach. However, the ENZA-p study has several caveats, including the fact that the patients are managed in a clinical trial setting instead of real-life clinical practice, raising concerns on the practical implementation of this treatment approach. Moreover, the ENZA-p study evaluated the concomitant use of enzalutamide and [^177^Lu]Lu-PSMA-617 in first-line mCRPC, instead of the currently licensed indication of post-taxane and post-ARPI mCRPC. Owing to the additive effect of enzalutamide and the generally better treatment responsiveness in an early disease stage, cautions should be exercised to extrapolate the outcomes of the interim imaging and adaptive dosing approach from ENZA-p to the current common practice of prescribing [^177^Lu]Lu-PSMA-617/analogue monotherapy in later-line mCRPC.

### 4.5 Part 5—Number of cycles

Respondents reached strong consensus on the recommendation of completing six cycles of [^177^Lu]Lu-PSMA-617/analogues in patients who have responded and who showed significant remaining uptake on PSMA PET after four cycles of treatment (Q20). With regard to patients who have no remaining uptake after four cycles of [^177^Lu]Lu-PSMA-617/analogues (Q21), no consensus was reached for any answer option; however, a combined 84% of the respondents thought that at least selected patients should complete six cycles of treatment.

**Q20**. In the majority of patients with response (PSA and/or clinical and/or radiological) to [^177^Lu]Lu-PSMA-617/analogues after four cycles and significant remaining uptake, 90% of the respondents recommended completion of six cycles in the majority of patients, whilst 10% recommended completion of six cycles only in selected patients. There were three abstentions. (Strong consensus for completion of six cycles in the majority of patients)

**Q21**. In the majority of patients with response (PSA and/or clinical and/or radiological) to [^177^Lu]Lu-PSMA-617/analogues after four cycles and no remaining uptake, 53% of the respondents recommended completion of six cycles only in selected patients, 31% recommended completion of six cycles in the majority of patients, and 16% did not recommend completion of six cycles. There were five abstentions. (No consensus for any answer option)

For Q20, both the APCCC 2024 panel members (64) and local experts had consistent opinions, achieving consensus (76%) and strong consensus (90%), respectively, on recommending the completion of six cycles in the majority of patients with response (PSA and/or clinical and/or radiological) to [^177^Lu]Lu-PSMA-617/analogues after four cycles and significant remaining uptake.

For Q21, most (57%) of the APCCC 2024 panel members did not recommend completion of six cycles in the majority of patients with response (PSA and/or clinical and/or radiological) to [^177^Lu]Lu-PSMA-617/analogues after four cycles and no remaining uptake (64). In comparison, most (53%) of the local experts recommended completion of six cycles only in selected patients compared with 25% of the APCCC panellists choosing this option.

The approach of pausing after the fourth cycle and reconsidering whether to continue with [^177^Lu]Lu-PSMA-617/analogues stemmed from the VISION trial (43). This design of the VISION trial indicated that the presence of significant remaining uptake supports the continuation of the remaining cycles of [^177^Lu]Lu-PSMA-617, whilst further evidence is required to justify completion of all six cycles in patients without remaining uptake after cycle 4. In addition, the term “significant remaining uptake” is yet to be delineated.

### 4.6 Part 6—General RLT questions

Part 6 of the questionnaire collected the perspectives of the specialists on combination therapy with ^177^Lu-PSMA plus an ARPI, extrapolation of the clinical data on [^177^Lu]Lu-PSMA-617 to analogues with other PSMA ligands, and the criteria for re-treatment with [^177^Lu]Lu-PSMA-617/analogues. No consensus was reached for any question in this part.

**Q22**. For the majority of patients treated with [^177^Lu]Lu-PSMA-617/analogues for mCRPC post-ARPI therapy and post-chemotherapy, 62% of the respondents did not recommend [^177^Lu]Lu-PSMA-617/analogues + ARPI, 22% recommended continuation with the current or previous ARPI, and 16% recommended combination with alternate ARPI. There were six abstentions. (No consensus for any answer option)

Most respondents (62%) did not recommend combination therapy. One panellist noted that, upon progression on or after ARPI, it is reasonable to omit ARPI therapy because the risk of side effects and treatment cost might outweigh the modest clinical benefits of combination therapy.

Post-hoc analyses of VISION (84) showed that 54.8% of the [^177^Lu]Lu-PSMA-617 group received concomitant ARPIs and that this patient subgroup had numerically longer median OS (17.8 vs. 12.4 months) and rPFS (10.2 vs. 8.5 months) compared with the subgroup that did not receive concomitant ARPIs. In the phase II randomised ENZA-p study, [^177^Lu]Lu-PSMA-617 plus enzalutamide significantly improved the PSA PFS compared with enzalutamide alone as the first-line treatment of mCRPC (61). Although these data suggest that [^177^Lu]Lu-PSMA-617/analogues plus an ARPI could yield enhanced anticancer activity, the efficacy and safety of such combination in the first- and later-line settings of mCRPC should be further investigated.

**Q23**. With regard to the data generated by PSMAfore and VISION with [^177^Lu]Lu-PSMA-617, 46% of the respondents considered that these data can be extrapolated to [^177^Lu]Lu-PSMA-I&T only, 35% considered that these data cannot be extrapolated to any alternate PSMA ligand, and 19% considered that these data can be extrapolated to any alternate PSMA ligand. There were 17 abstentions. (No consensus for any answer option)

The highest level of evidence for PSMA-targeted RLT was derived from studies on [^177^Lu]Lu-PSMA-617. The randomised phase III VISION trial (43) has led to the worldwide regulatory approval of the first PSMA-targeted RLT (41, 42, 85). [^177^Lu]Lu-PSMA-617 has also been studied in multiple randomised trials, such as TheraP (vs. cabazitaxel for later-line mCRPC) (73), ENZA-p (in combination with enzalutamide vs. enzalutamide alone for first-line mCRPC) (61), and UpFrontPSMA (sequential use followed by docetaxel vs. docetaxel alone for de novo mHSPC) (62). Additional studies on [^177^Lu]Lu-PSMA-617 are underway in the mHSPC (86) and oligometastatic PCa settings (87).

On the other hand, several phase III studies on other [^177^Lu]Lu-PSMA RLTs are ongoing, such as [^177^Lu]Lu-PNT2002 in the SPLASH trial and [^177^Lu]Lu-PSMA-I&T in the ECLIPSE trial (88, 89). Recent results from the SPLASH trial revealed that, in mCRPC patients with PSMA-avid lesions and prior ARPI use, [^177^Lu]Lu-PNT2002 at 6.8 GBq every 8 weeks for up to four cycles significantly improved the median rPFS (9.5 vs. 6.0 months; HR = 0.71, 95%CI = 0.55–0.92, p = 0.0088) compared with alternate ARPI therapy (88). Of patients on [^177^Lu]Lu-PNT2002, 38.2% had a complete or partial response, and 37.2% had a side effect of dry mouth (88). However, the efficacy of [^177^Lu]Lu-PNT2002 appears to be less promising than that of [^177^Lu]Lu-PSMA-617, as shown in the PSMAfore trial (56). With the setting and follow-up period being similar to SPLASH, the second interim analysis of PSMAfore demonstrated that [^177^Lu]Lu-PSMA-617 at 7.4 GBq every 6 weeks for up to six cycles significantly improved the median rPFS (12.0 vs. 5.59 months; HR = 0.43, 95%CI = 0.33–0.54, p < 0.0001) compared with ARPI therapy (56). In the [^177^Lu]Lu-PSMA-617 group, 50.7% had a complete or partial response, and 57.3% had dry mouth (56). Comparing SPLASH and PSMAfore, the lower dose and the longer interval of [^177^Lu]Lu-PNT2002 versus [^177^Lu]Lu-PSMA-617 appeared to reduce the treatment response (median rPFS = 9.5 vs. 12.0 months, HR = 0.71 vs. 0.43; rate of complete/partial response, 38.2% vs. 50.7%), with a modestly reduced rate of dry mouth (37.2% vs. 57.3%) (56, 88).

A combined 65% of the respondents considered that the current clinical data on [^177^Lu]Lu-PSMA-617 can be extrapolated to other PSMA ligands (all ligands, 19%; only PSMA-I&T, 46%) despite the distinct chemical structures of each ligand. In contrast, a majority (56%) of the APCCC 2024 panel members disagreed with such extrapolation (64). At the local roundtable discussion, a panellist pointed out that there is no direct comparison between PSMA-targeted RLTs. One panellist noted that the structural differences of these agents could lead to distinct profiles of metabolic stability and antibody affinity. In any case, the effects of these pharmacokinetic properties on clinical outcomes remain to be investigated.

**Q24**. In the disease course in patients who have previously responded to four or more cycles of [^177^Lu]Lu-PSMA-617/analogues, if the relevant PET criteria are met, 37% of the respondents recommended re-treatment with [^177^Lu]Lu-PSMA-617/analogues in those with response of >6 months, 24% recommended re-treatment with [^177^Lu]Lu-PSMA-617/analogues in all patients, 21% did not recommend re-treatment with [^177^Lu]Lu-PSMA-617/analogues, and 18% recommended re-treatment with [^177^Lu]Lu-PSMA-617/analogues in those with response of >12 months. There were five abstentions. (No consensus for any answer option)

In this area with scarce evidence, a combined 79% of the respondents recommended re-treatment with [^177^Lu]Lu-PSMA-617/analogues in patients with disease progression who previously responded to four or more cycles of this treatment over different durations of the prior response. At the roundtable discussion, the panellists speculated that the high proportion of votes for re-treatment was grounded on the rationale that patients are short of treatment options. For patients who had a long enough duration of response towards prior [^177^Lu]Lu-PSMA-617/analogues, re-treatment may be the only treatment option left. Most (59%) of the APCCC 2024 panellists voted to re-treat patients who showed a response duration of >6 months towards four or more cycles of [^177^Lu]Lu-PSMA-617/analogues (64), which was also the option chosen by most (37%) local experts. The 6-month interval between the initial treatment and re-treatment was derived from the evidence with docetaxel, which demonstrated a higher likelihood of clinical benefit among patients who have responded for longer than half a year (90). Accounting for the fact that 13 months have already lapsed at 6 months after the sixth cycle of [^177^Lu]Lu-PSMA-617/analogues, the APCCC 2024 expert panel even considered re-treatment at a shorter interval (64). More clinical data are warranted to determine the optimal interval between the initial course and re-treatment with [^177^Lu]Lu-PSMA-617/analogues.

In the clinical experience of the panellists, instead of the full course of four to six cycles, generally only two to three cycles of [^177^Lu]Lu-PSMA-617/analogues were administered in the re-treatment phase, which resulted in short-lived efficacy and acceptable toxicity. One panellist shared the observation that prior radium-223 treatment was associated with an elevated risk of grade 2/3 bone marrow toxicity when re-treated with [^177^Lu]Lu-PSMA-617/analogues. Nonetheless, the RALU study, a multicentre, retrospective, medical chart review of 133 patients reported no indication of impairment of the safety or effectiveness of [^177^Lu]Lu-PSMA-617/analogues after radium-223 (91).

In the phase II prospective LuPSMA study, among 50 patients with mCRPC who received up to four cycles of [^177^Lu]Lu-PSMA-617, 15 (30%) demonstrated sufficiently PSMA-avid disease and no discordant sites of disease on repeat [^68^Ga]Ga-PSMA-11 and [^18^F]FDG imaging upon biochemical progression (92). These patients received further [^177^Lu]Lu-PSMA-617, commencing a median of 359 days after study enrolment for up to a median of two cycles (range = 1–5). Most (11/15, 73%) of the patients achieved a PSA decline of ≥50%, representing a high response rate towards additional cycles of [^177^Lu]Lu-PSMA-617 upon progression. Treatment-emergent adverse events were similar to those of the initial therapy (92). The limitations of this study included a single-arm, single-centre design with a small number of patients. One panellist noted that patients with a lower risk of disease progression would be more likely to receive re-treatment with [^177^Lu]Lu-PSMA-617.

### 4.7 Part 7—Impaired bone marrow function

Patients with advanced PCa often exemplify impaired bone marrow function due to prior treatment and bone metastasis (93), which in turn influences treatment decision. The two questions in Part 7 asked the specialists about their recommendation for patients with impaired bone marrow function who received prior ARPI with (Q26) or without (Q25) prior chemotherapy. No consensus was reached for both questions.

**Q25**. In the majority of patients with mCRPC (no DDR alteration) and relevant impaired bone marrow function (haemoglobin <90 g/L and/or neutrophils <1.5 × 10^9^/L and/or platelets <100 × 10^9^/L), after receiving ARPI therapy, 24.5% of the respondents recommended [^177^Lu]Lu-PSMA-617/analogues at reduced activity, 24.5% recommended alternate ARPI, 19% recommended weekly docetaxel, 16% recommended [^177^Lu]Lu-PSMA-617/analogues at standard activity, and 16% recommended best supportive care. There were six abstentions. (No consensus for any answer option)

**Q26**. In the majority of patients with mCRPC (no DDR alteration) and relevant impaired bone marrow function (haemoglobin <90 g/L and/or neutrophils <1.5 × 10^9^/L and/or platelets <100 × 10^9^/L), after receiving ARPI therapy and docetaxel, 31% of the respondents recommended [^177^Lu]Lu-PSMA-617/analogues at reduced activity, 28% recommended alternate ARPI, 19% recommended best supportive care, 14% recommended [^177^Lu]Lu-PSMA-617/analogues at standard activity, 5% recommended weekly or 2-weekly cabazitaxel, and 3% recommended radium-223. There were seven abstentions. (No consensus for any answer option)

With reference to the Hong Kong Prescribing Information of [^177^Lu]Lu-PSMA-617, patients with grade 2 bone marrow suppression (haemoglobin <100–80 g/L and/or neutrophils <1.5–1.0 × 10^9^/L and/or platelets <75.0–50.0 × 10^9^/L) should resume [^177^Lu]Lu-PSMA-617 at standard activity upon improvement to grade 1 or baseline, whilst those with grade 3 bone marrow suppression (haemoglobin <80 g/L and/or neutrophils <1.0–0.5 × 10^9^/L and/or platelets <50.0–25.0 × 10^9^/L) should resume [^177^Lu]Lu-PSMA-617 at reduced activity upon improvement to grade 1 or baseline, under the current licensed indication of post-ARPI and post-taxane PSMA-positive mCRPC (94). Moreover, the same thresholds are also applicable to baseline values at the time of initiation with [^177^Lu]Lu-PSMA-617 (94).

For Q25, most (49%) of the APCCC 2024 panel members voted on weekly docetaxel for patients with relevant impaired bone marrow function after prior ARPI therapy, whereas only 19% of local experts chose this option (64). On the other hand, 25% and 24% of local experts voted on [^177^Lu]Lu-PSMA-617/analogues at reduced activity and alternate ARPI, respectively. In comparison, only 12% of the APCCC panellists chose each of these options (64).

For Q26, among patients previously treated with ARPI and docetaxel, 8% of the APCCC 2024 panel members chose cabazitaxel 3-weekly (64), whilst none of the local experts did. Moreover, no APCCC 2024 panel members voted for radium-223 (64), whilst 3% of the local experts recommended this treatment option. Treatment with [^177^Lu]Lu-PSMA-617/analogues at reduced activity was the most popular option among both APCCC panellists (27%) and local experts (31%).

At the roundtable discussion, several experts noted that, according to the VISION study protocol, [^177^Lu]Lu-PSMA-617 therapy should not be used in patients with relevant impaired bone marrow function (43). They added that bone marrow toxicity is also a major barrier to chemotherapy and targeted therapies; therefore, best supportive care is often chosen for the patients concerned. However, one panellist suggested that low-dose chemotherapy may be an option in selected patients, such as those who have no prior exposure to chemotherapy, an acceptable performance status, and satisfactory organ function. Given that weekly docetaxel is still relatively toxic to patients with borderline bone marrow function, reduced-dose docetaxel may be the preferred option when the bone marrow suppression is disease-related, which suggests intolerability to radiopharmaceuticals, i.e., [^177^Lu]Lu-PSMA-617/analogues and radium-223. On the other hand, another panellist indicated that reduced-activity [^177^Lu]Lu-PSMA-617/analogues adapted to patients’ organ functions appear to be an intriguing option; however, more clinical evidence is required to support such practice. Practical logistics could form an additional barrier to administering [^177^Lu]Lu-PSMA-617/analogues at lowered activity.

### 4.8 Part 8—Impaired renal function

[^177^Lu]Lu-PSMA-617/analogues are primarily eliminated by the renal route (94). Questions of potential nephrotoxicity are based on concerns of renal tubular PSMA expression and the resultant radiopharmaceutical retention during RLT (95). Impaired renal function is associated with possible retention of the radiopharmaceutical that leads to increased exposure (96). However, there is still a lack of evidence proving the clinically significant nephrotoxicity of [^177^Lu]Lu-PSMA-617/analogues in patients with impaired renal function (95). Towards the end of the questionnaire, experts were asked about the effect of renal function derangement on treatment decision-making for patients with advanced PCa.

**Q27**. In the majority of patients with mCRPC (no DDR alteration) progressing on or after an ARPI and with impaired renal function [glomerular filtration rate (GFR) = 30–49 mL/min], 37% of the respondents recommended docetaxel, 29% recommended [^177^Lu]Lu-PSMA-617/analogues at reduced activity, 20% recommended [^177^Lu]Lu-PSMA-617/analogues at standard activity, and 14% recommended alternate ARPI. There were eight abstentions. (No consensus for any answer option)

Although no consensus was reached, a combined 49% of the respondents recommended [^177^Lu]Lu-PSMA-617/analogues, either at standard activity (20%) or at reduced activity (29%) in patients with renal impairment (GFR = 30–49 mL/min). A substantially lower percentage (a combined 18%) of the APCCC 2024 panel members recommended [^177^Lu]Lu-PSMA-617/analogues in the same question (64). On the other hand, 73% of the APCCC 2024 panel members recommended docetaxel.

Nevertheless, with reference to the Hong Kong Prescribing Information of [^177^Lu]Lu-PSMA-617, patients with a confirmed grade 2 serum creatinine increase (>1.5–3.0× baseline; >1.5–3.0× upper limit of normal) or a confirmed creatinine clearance (CrCl) <50 mL/min calculated with Cockcroft–Gault with actual body weight should resume [^177^Lu]Lu-PSMA-617 at standard activity upon improvement, whilst those with grade 3 confirmed ≥40% serum creatinine increase from baseline or a confirmed >40% decrease from baseline CrCl should resume [^177^Lu]Lu-PSMA-617 at reduced activity upon improvement or return to baseline (94). Patients with recurrent renal toxicity (grade 3 or higher) should permanently discontinue [^177^Lu]Lu-PSMA-617 (94). Moreover, no dose adjustment is recommended for patients with mild-to-moderate renal impairment with baseline CrCl ≥50 mL/min using Cockcroft–Gault (94). Treatment with [^177^Lu]Lu-PSMA-617 is not recommended in patients with moderate-to-severe renal impairment with baseline CrCl <50 mL/min or end-stage renal disease, as the pharmacokinetic profile and the safety of [^177^Lu]Lu-PSMA-617 have not been studied in these patients (94).

In the panel discussion, several panellists noted that, despite elimination via the renal route, [^177^Lu]Lu-PSMA-617/analogues are not necessarily nephrotoxic. A post-hoc analysis of the prospective REALITY study showed that, among 22 mCRPC patients with impaired renal function (mean GFR = 45.0 ± 10.7 mL/min) who received two to six cycles of [^177^Lu]Lu-PSMA-617 (median dose = 6.5 GBq/cycle; 10/22 patients completed six cycles), the end-of-treatment GFR (54.1 ± 16.7 mL/min) was significantly higher than the baseline GFR (p = 0.016) (95). A vast majority (21/22) of patients showed no significant reduction in GFR at follow-up assessments (6, 9, and 12 months) (95). The per-cycle (p = 0.605) or cumulative (p = 0.132) administered activity was not correlated with changes in the GFR (95). Therefore, the authors concluded that the nephrotoxic potential of [^177^Lu]Lu-PSMA-617 may be overestimated and that patients should not be definitely excluded from PSMA-RLT due to renal impairment (95).

One panellist added that there is no evidence that reducing the dose of [^177^Lu]Lu-PSMA-617/analogues alleviates the nephrotoxicity whilst maintaining efficacy. Several panellists shared that they preferred addressing the underlying renal conditions that lead to deranged renal function, followed by initiating [^177^Lu]Lu-PSMA-617/analogues at standard activity.

## 5 Discussion and conclusion

Although mCRPC remains incurable, a wide range of systemic therapies, including chemotherapy, ARPIs, bone-targeted radionuclides, targeted therapies, and RLT, have substantially improved the survival outcomes. Notably, optimal treatment sequencing is crucial as patients with mCRPC often require multiple lines of treatment, and the number of patients receiving therapy decreases per subsequent treatment lines, possibly due to death or deterioration in physical condition that induces ineligibility to treatment. RLT, such as [^177^Lu]Lu-PSMA-617/analogues, is a novel treatment for mCRPC. To optimise the clinical application of this agent, the HKSUO designed a questionnaire to survey PCa experts in Hong Kong on the clinical utility of PSMA-targeted RLT. A total of 43 respondents voted on 27 questions, followed by a roundtable discussion meeting of a panel of 13 experts who discussed the questionnaire results and provided further insights into specific questions.

Based on the questions for which consensus or strong consensus was reached, [^177^Lu]Lu-PSMA-617/analogues are recommended for patients with PSMA imaging-positive mCRPC who meet the relevant PET criteria for [^177^Lu]Lu-PSMA-617/analogues with no actionable molecular alterations, have received one line of ARPI therapy and one line of taxane-based chemotherapy, or have received one line of ARPI therapy and are unfit for chemotherapy, including symptomatic patients eligible for radium-223 regardless of the extent of visceral metastasis. In addition, strong consensus was attained on the completion of six cycles of [^177^Lu]Lu-PSMA-617/analogues among patients who responded to [^177^Lu]Lu-PSMA-617/analogues and who show significant remaining uptake on PSMA PET after four cycles. With regard to the evaluation of patient eligibility for [^177^Lu]Lu-PSMA-617/analogues, consensus was reached on employing [^68^Ga]Ga-PSMA-11 as the primary PSMA PET radioligand, as well as to add [^18^F]FDG PET selectively in equivocal cases.

Notably, no consensuses were reached for most (17/27, 63%) of the questions, suggesting that a variety of uncertainties regarding RLT, particularly the optimal approach for response monitoring, as well as its application in patients with deranged organ functions, warrant further research. No consensus was reached with regard to the optimal treatment regimens for post-ARPI, chemotherapy-fit patients who are positive for PSMA and have molecular alterations, including HRR gene mutations (except *BRCA1/2*), dMMR, or MSI-high, suggesting that targeted therapies may not necessarily precede PSMA-RLT for patients with molecular alterations, especially in the post-taxane setting.

In conclusion, the literature review, questionnaire results, and expert opinions are anticipated to collectively provide a practical guide for clinicians to navigate recent therapeutic advancements and the role of novel treatment modality, PSMA-targeted RLT, in the treatment of mCRPC.
